# A novel approach to depression detection using POV glasses and machine learning for multimodal analysis

**DOI:** 10.3389/fpsyt.2025.1720990

**Published:** 2025-11-10

**Authors:** Hakan Kayış, Murat Çelik, Vildan Çakır Kardeş, Hatice Aysima Karabulut, Ezgi Özkan, Çınar Gedizlioğlu, Burcu Özbaran, Nuray Atasoy

**Affiliations:** 1 Department of Child and Adolescent Psychiatry, Faculty of Medicine, Zonguldak Bülent Ecevit University, Zonguldak, Türkiye; 2 Independent Researcher, Ankara, Türkiye; 3 Department of Psychiatry, Faculty of Medicine, Zonguldak Bülent Ecevit University, Zonguldak, Türkiye; 4 Department of Computer Engineering, İzmir University of Economics, Izmir, Türkiye; 5 Department of Child and Adolescent Psychiatry, Faculty of Medicine, Ege University, Izmir, Türkiye

**Keywords:** major depressive disorder, machine learning, multimodal analysis, wearable technology, point-of-view glasses, artificial intelligence, computer vision

## Abstract

**Background:**

Major depressive disorder (MDD) remains challenging to diagnose due to its reliance on subjective interviews and self-reports. Objective, technology-driven methods are increasingly needed to support clinical decision-making. Wearable point-of-view (POV) glasses, which capture both visual and auditory streams, may offer a novel solution for multimodal behavioral analysis.

**Objective:**

This study investigated whether features extracted from POV glasses, analyzed with machine learning, can differentiate individuals with MDD from healthy controls.

**Methods:**

We studied 44 MDD patients and 41 age/sex-matched HCs (18–55 years). During semi-structured interviews, POV glasses recorded video and audio data. Visual features included gaze distribution, smiling duration, eye-blink frequency, and head movements. Speech features included response latency, silence ratio, and word count. Recursive feature elimination was applied. Multiple classifiers were evaluated, and the primary model—ExtraTrees—was assessed using leave-one-out cross-validation.

**Results:**

After Bonferroni correction, smiling duration, center gaze and happy face duration showed significant group differences. The multimodal classifier achieved an accuracy of 84.7%, sensitivity of 90.9%, specificity of 78%, and an F1 score of 86%.

**Conclusions:**

POV glasses combined with machine learning successfully captured multimodal behavioral markers distinguishing MDD from controls. This low-burden, wearable approach demonstrates promise as an objective adjunct to psychiatric assessment. Future studies should evaluate its generalizability in larger, more diverse populations and real-world clinical settings.

## Introduction

Major depressive disorder (MDD) is one of the leading causes of disability worldwide, affecting more than 280 million people across different age groups and cultural backgrounds (1). Beyond its profound impact on quality of life, MDD contributes substantially to the global burden of disease, ranking among the top contributors to years lived with disability (2). Early and accurate detection of MDD is therefore critical, as timely interventions can significantly improve treatment outcomes and reduce long-term socioeconomic costs (3).

Despite this urgency, current diagnostic approaches rely primarily on clinical interviews and self-report questionnaires such as the Diagnostic and Statistical Manual of Mental Disorders, Fifth Edition (DSM-5) criteria and the Beck Depression Inventory (BDI). While widely adopted, these tools are inherently subjective, susceptible to recall bias, and may vary in reliability across clinical contexts (4, 5). Therefore, there is a growing demand for objective, reproducible, and scalable biomarkers that can complement traditional psychiatric assessments.

In recent years, advances in AI and ML have enabled automated extraction of behavioral and affective features from visual and auditory data. Visual modalities including facial-expression analysis using the Facial Action Coding System (FACS), gaze tracking, and head-movement dynamics (6–8) have shown promise in distinguishing individuals with depression from healthy controls; depression is often characterized by slower head movements, reduced smiling, and restricted affect (8, 9). Auditory and linguistic modalities have also been explored, with features such as reduced speech intensity, monotony, increased jitter, and less phonetic variability associated with depressive symptoms (10–12). Speech-based models using ML classifiers have demonstrated moderate to high accuracy in detecting MDD, supporting the potential of audio features as digital biomarkers (13).

While both visual and auditory markers have shown promise individually, recent studies emphasize the advantage of multimodal approaches that integrate multiple channels of information (14–16). By combining complementary data sources, multimodal models tend to achieve superior accuracy and robustness compared to unimodal systems. However, most existing research relies on stationary cameras and webcams in laboratory environments, which may constrain ecological validity and fail to capture naturalistic interactions. To address these limitations and realize the benefits of multimodality in real clinical settings, clinician-worn point-of-view (POV) glasses can be used to unobtrusively acquire multimodal signals during psychiatric interviews without altering their natural flow. Because the camera is mounted on the clinician rather than the patient, no patient-worn hardware is required, thereby avoiding comfort and adherence burdens and reducing reactivity (Hawthorne effects) associated with conspicuous equipment. The egocentric, face-aligned vantage point preserves authentic clinical context—typical conversational distance, spontaneous posture, and genuine turn-taking—so that gaze behavior, affect, and speech timing are recorded as they naturally occur. In practice, this configuration yields facial footage with fewer off-axis artifacts than room-mounted webcams and minimizes self-presentation bias, thereby enhancing ecological validity while keeping the encounter clinically routine.

A critical gap in the literature concerns the use of wearable devices to capture multimodal behavioral data in more natural settings. Point-of-view (POV) glasses, equipped with front-facing cameras and microphones, provide a unique vantage point by recording interactions directly from the participant’s perspective. This technology has the potential to overcome limitations of fixed-camera setups, offering a more ecological and unobtrusive method to assess behavioral patterns relevant to MDD. To date, only a limited number of studies have examined the utility of POV glasses in psychiatric research, and none have systematically evaluated their role in multimodal ML frameworks for depression detection.

The present study addresses this gap by investigating whether multimodal features extracted from POV glasses can be used to differentiate individuals with MDD from healthy controls. Specifically, visual features (e.g., gaze distribution, smiling duration, eye-blink frequency, and head movements) and speech features (egg, response latency, silence ratio, and word count) were analyzed within a machine learning framework.

We hypothesized that (1) multimodal behavioral markers (gaze, affective expressions, eye-blink patterns, head movements, and speech-derived features) would differ between groups, and (2) a classifier trained on these features would accurately distinguish MDD from controls. By introducing a wearable and low-burden method for capturing behavioral data, this study seeks to advance the development of objective and scalable tools to support clinical diagnosis of depression.

## Methods

### Study design

This study employed an observational, cross-sectional design to evaluate behavioral and affective markers of major depressive disorder (MDD) using multimodal data captured with wearable POV glasses. During semi-structured clinical interviews, the interviewer (a trained clinician) wore POV glasses equipped with a front-facing camera and an integrated microphone. This setup enabled the unobtrusive recording of participants’ visual and auditory behaviors from the clinician’s natural perspective, such as facial expressions, gaze patterns, eye-blink dynamics, and speech characteristics. All interviews were conducted in a standardized indoor environment to ensure consistency across participants, with uniform lighting and recording conditions.

### Recruitment

Participants were recruited from the Department of Psychiatry at Zonguldak Bülent Ecevit University Faculty of Medicine. The study consisted of two groups: patients diagnosed with major depressive disorder (MDD) and healthy controls.

Patients in the MDD group were consecutively enrolled among individuals presenting to the psychiatry outpatient clinic. The diagnostic process followed a structured two-step procedure: first, a psychiatric resident conducted the Structured Clinical Interview for DSM-5 Disorders (SCID-5), and subsequently, the diagnosis was confirmed by a board-certified psychiatrist with over 20 years of clinical experience. Healthy controls were recruited on a voluntary basis from the community and hospital staff and reported no current or past psychiatric disorders.

Inclusion criteria for both groups were: (1) age between 18 and 55 years and (2) willingness to participate in the study. Additionally, the MDD group required (3) a DSM-5 diagnosis of major depressive disorder confirmed through SCID-5 and (4) a Beck Depression Inventory (BDI) score ≥20. Healthy controls were required to score below the clinical threshold on the BDI. Within the MDD group, BDI severity distribution was as follows: moderate (20–28), n=28; severe (29–63), n=16, classified according to BDI thresholds for the Turkish adult population (17).

Exclusion criteria were: (1) history of neurological disorders, (2) strabismus or severe visual/hearing impairments that could interfere with audiovisual analysis, (3) cosmetic procedures such as botulinum toxin (Botox) injections within the past 6 months, (4) current or recent use of psychiatric medications, (5) comorbid bipolar disorder in the patient group, and (6) substance use disorder within the previous 12 months.

A total of 97 individuals were initially recruited for the study, including 50 patients with MDD and 47 healthy controls. However, data from several participants were excluded due to recording quality issues. In the MDD group, audio data from 2 participants could not be analyzed because of excessive background noise, and video data from 3 participants were excluded due to poor recording quality or excessive movement. In the control group, 4 participants were excluded for the latter reason. Furthermore, in 1 patient and 2 control participants, interaction data could not be obtained due to technical failures during the recording process. After these exclusions, the final sample comprised 85 participants: 44 patients with MDD and 41 healthy controls. The two groups were matched for age and sex, with no significant demographic differences between them.

**Figure 1:**
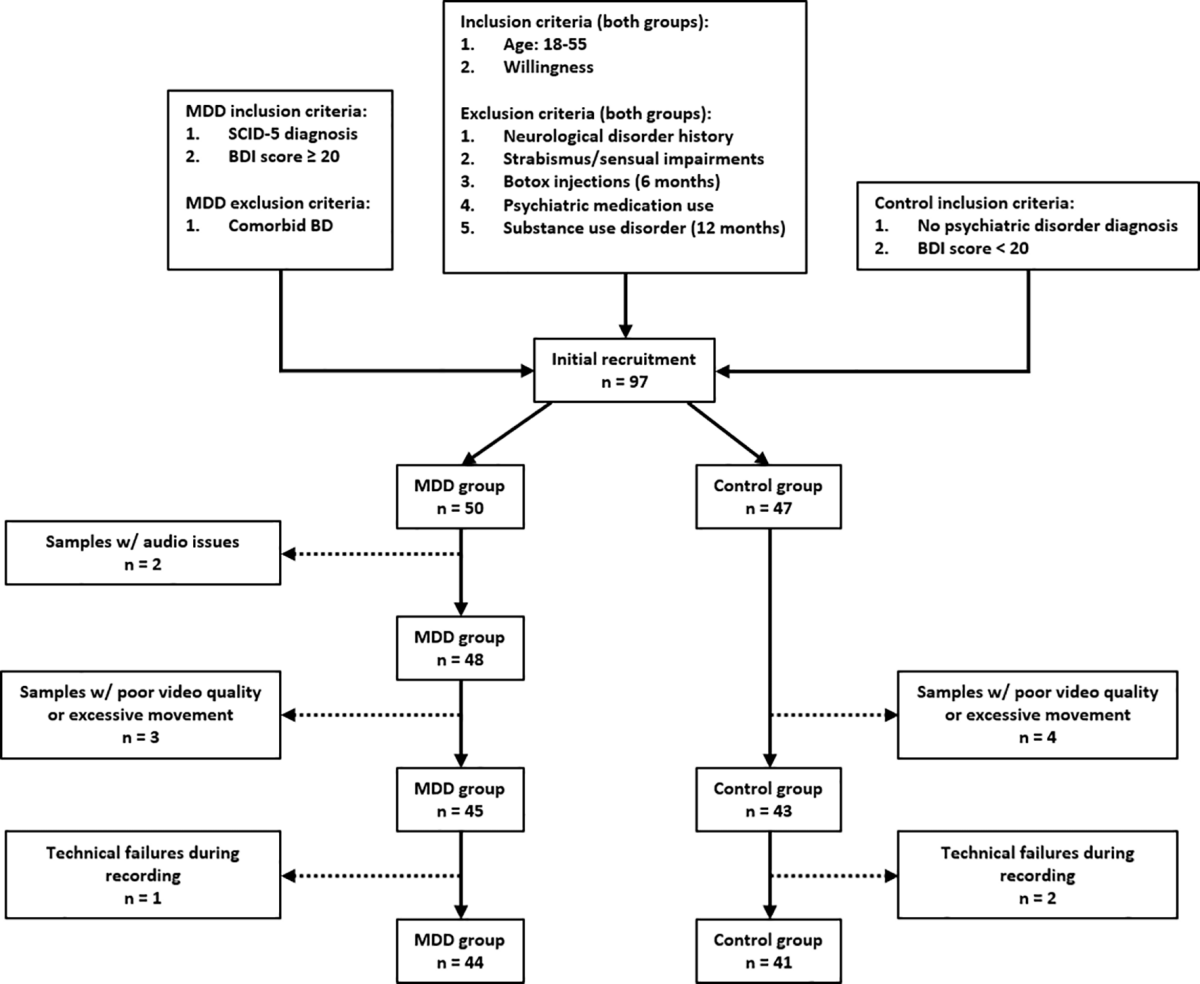
Participant flow diagram for inclusion and exclusion across eligibility, enrollment, and analysis.

A total of 97 individuals were initially recruited for the study, including 50 patients with MDD and 47 healthy controls. However, data from several participants were excluded due to recording quality issues. Short video dropouts (≤2 consecutive frames at 30 FPS) were corrected by linear interpolation; segments with ≥ 10% missing frames or severe artifacts (e.g., heavy motion blur, or occlusion) were excluded (excluded participants: n=1 MDD, n=2 HC). At the participant level, recordings with ≥ 20% missing data in any domain were excluded (18) from the corresponding group (inferential) analyses (excluded participants: video-based features: n=3 MDD, n=4 HC; speech features: n=2 MDD), yielding a complete-case dataset for hypothesis testing. After these exclusions, the final sample comprised 85 participants: 44 patients with MDD and 41 healthy controls. The two groups were matched for age and sex, with no significant demographic differences between them. For the machine-learning pipeline, residual missing entries <20% (per feature × participant) were imputed within each training fold only (median for continuous, mode for categorical), and the fitted imputer was applied to the held-out participant to avoid information leakage. Subsequent statistical tests and ML modeling were performed on the resulting datasets as specified above. A flow diagram of the inclusion-exclusion criteria can be seen in **Figure 1**.

### Data collection

All participants underwent a semi-structured clinical interview while seated face-to-face with the clinician. During each interview, the clinician wore POV glasses equipped with a front-facing high-definition camera (1080p resolution at 30 frames per second) and an integrated microphone (19). This setup enabled the unobtrusive audiovisual recording of participants’ verbal and nonverbal behaviors from the natural perspective of the interviewer.

The interview protocol consisted of four open-ended questions designed to elicit spontaneous speech, emotional expression, and natural interaction. The questions were as follows:

1. How have you been feeling recently? Could you describe this in detail?
2. Can you describe a typical day, from the moment you wake up in the morning until you go to bed at night?
3. Can you describe a positive moment that made you feel good in detail?
4. Can you describe a negative moment that made you feel bad in detail?

Each response lasted a minimum of 30 seconds, yielding sufficient audiovisual material for subsequent computational analysis. Interviews were conducted in a standardized clinical room with controlled environmental conditions. The distance between participant and clinician was maintained at approximately 100 cm, and ambient illumination was kept within 400–600 lux to minimize variability in facial feature detection. Background noise was reduced to ensure high-quality audio capture.

Following data collection, video recordings were prepared for analysis by extracting 30-second segments corresponding to each interview question. This step was performed using Movavi Video Editor (Version 22.0, Movavi, 2023), ensuring that only the relevant portions of the recordings were retained for further processing (20). Importantly, the researcher responsible for analyzing the video recordings was blinded to the diagnostic status of the participants to minimize potential bias. All recordings were securely stored in encrypted format and were accessible only to authorized members of the research team.

### Feature extraction

A comprehensive set of audiovisual features was extracted from the recordings. The measured parameters included: duration of gaze directed at the interviewer (center), time spent looking to the right and left, smiling duration, simultaneous occurrence of forward gaze with smiling (social smiling), duration of neutral and happy facial expressions, number and duration of blinks, eye openness, total head movement, rapid head movements, response latency, silence ratio, and word count. For each participant, these parameters were measured across four 30-second video segments corresponding to the interview questions. To obtain a single representative score per feature, values from the four segments were averaged. This procedure minimized variability across questions and provided a stable estimate of each participant’s typical behavioral pattern.

**Figure 2:**
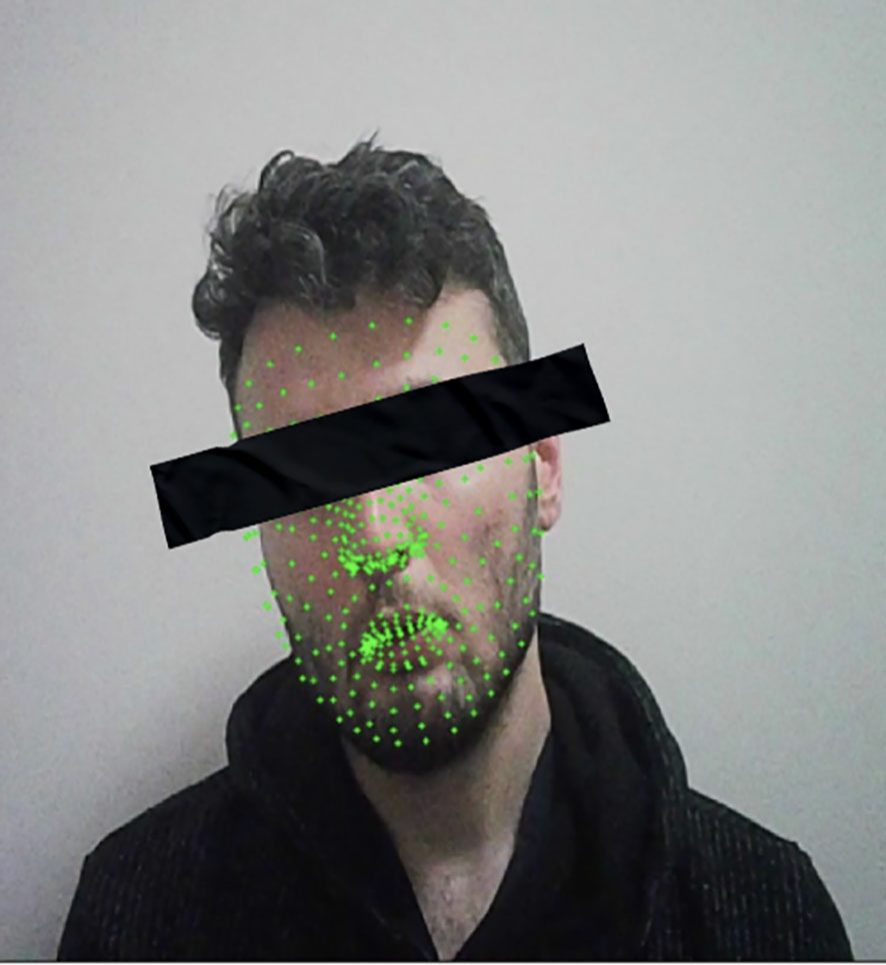
Example frame demonstrating *MediaPipe FaceMesh* feature detection used in this study. Green facial landmarks represent automatically identified key points across the participant’s face, which were utilized to calculate head pose, eye aspect ratio, and blink metrics.

Facial landmarks, head movements, eye openness, and blink-related parameters were extracted using MediaPipe. MediaPipe is an open-source library that provides high-accuracy and accessible methods for detecting facial and body landmarks in video data (21–23). In particular, its FaceMesh module enables the estimation of 3D head pose (pitch, yaw, roll) from 2D video.(**Figure 2**) Based on these angles, rapid head movements were defined as frame-to-frame changes greater than 5° on any axis, while total head movement was calculated as the cumulative sum of absolute angular changes across all three axes.

**Figure 3:**
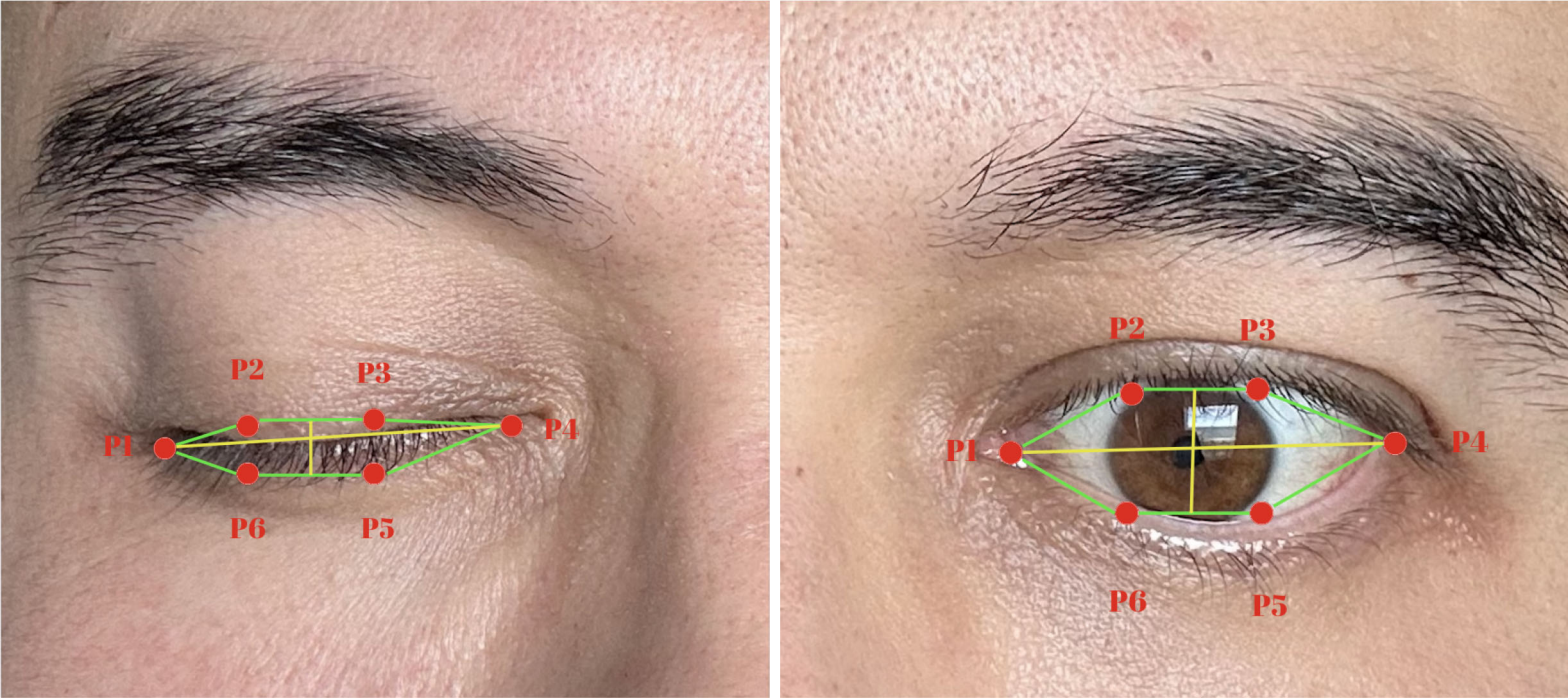
Illustration of the *Eye Aspect Ratio (EAR)* measurement used for blink detection. Six key landmarks (P1–P6) define the geometric relationships between the upper and lower eyelids. EAR is computed as the ratio of the vertical to horizontal distances between these points, allowing automated identification of eye-opening and eye-closing states across video frames.


Eye openness and blink metrics were quantified using the Eye Aspect Ratio (EAR) (24). EAR is a geometric index that determines whether the eye is open or closed by using six landmarks around the eye (p1–p6), measured with MediaPipe. (**Figure 3**) It is defined as:

EAR = (d(p2,p6) + d(p3,p5)/2))/d(p1,p4)

where *d(·)* represents the Euclidean distance between two points. A blink was identified when EAR <0.2, a threshold commonly reported in the literature. For analysis, an EAR value greater than 0.20 was taken to indicate that the eyes were open. Eye openness was therefore calculated as the mean EAR value across all frames exceeding this threshold, representing the average degree of eye opening. Blink count was defined as the number of discrete sequences in which the EAR dropped below 0.20 and subsequently rose above it again, each such cycle being counted as one blink. Blink duration was defined as the average length of these sequences, calculated by dividing the total number of frames with EAR < 0.20 by the total number of blinks in the recording.

**Figure 4:**
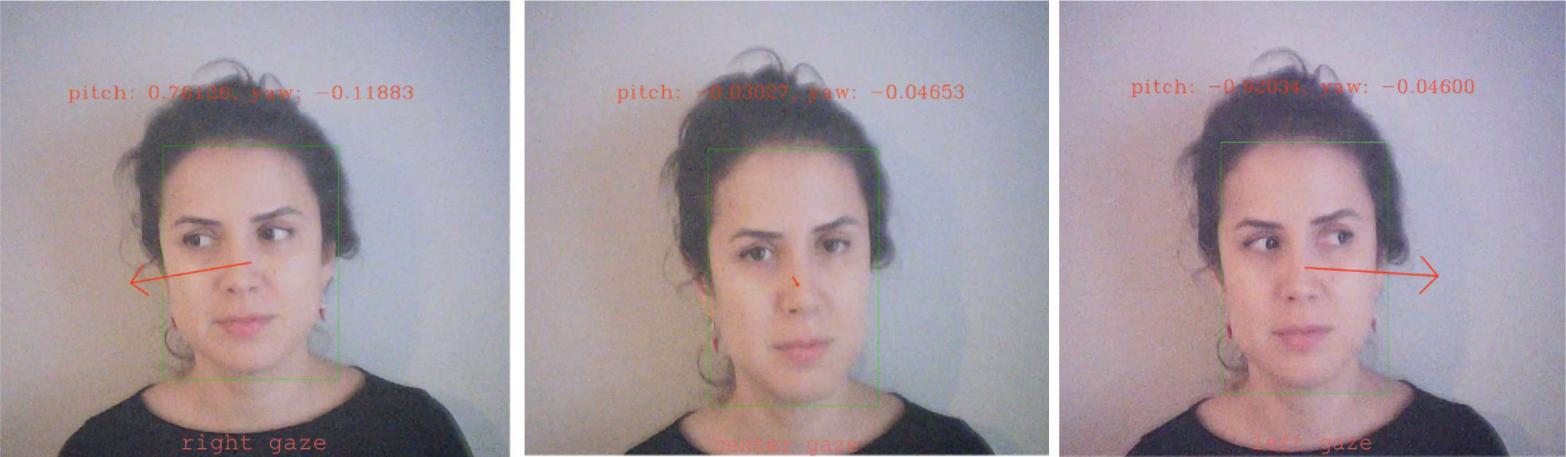
Examples of gaze direction estimation using *L2CS-Net*. The model predicts yaw and pitch angles from facial landmarks to classify gaze orientation into right, center, and left directions. The arrows illustrate the estimated gaze vectors, and angle values (pitch, yaw) are displayed for each frame.

Eye gaze direction was estimated using L2CS-Net, a deep convolutional neural network developed for fine-grained gaze estimation in unconstrained environments. L2CS-Net employs a dual-branch architecture in which yaw and pitch angles are predicted independently through a combination of classification and regression. Discretized angle bins are used for classification via a softmax cross-entropy loss, while continuous estimates are refined through regression using mean squared error. This hybrid loss design allows the model to achieve both categorical robustness and fine-grained precision (25). In our study, gaze direction was categorized into *left, right*, and *center* classes, (**Figure 4**) with aggregated measures calculated for each interview segment. Raw yaw and pitch values are output in radians (−π to +π). In our recordings, due to the camera/model axis convention, the horizontal component of gaze aligned with the model’s *pitch* (pitch > 0 → right, pitch < 0 → left), whereas *yaw* reflected vertical orientation and was not used for class assignment. Accordingly, frames were classified as right if pitch ≥ +0.17 rad (≈10°), left if pitch ≤ −0.17 rad, and center if |pitch| < 0.17 rad. To avoid confounds from extreme vertical gaze, we further restricted analyses to frames with |yaw| ≤ 0.35 rad (~20°); frames outside this range were marked invalid and excluded from aggregation.

Emotional expressions were analyzed using the Facial Action Coding System (FACS) (Ekman & Friesen, 1978), which decomposes facial expressions into individual muscle movements referred to as Action Units (AUs) (26). For example, AU12 corresponds to the contraction of the zygomaticus major muscle, which produces a smile, while combinations of AUs can represent complex emotions such as happiness or sadness. Happiness was identified by the co-activation of AU6 (orbicularis oculi, producing eye constriction) and AU12, a combination that is commonly used in the literature to index genuine positive affect (Duchenne smiles). Neutral emotion was defined as the absence of major expressive AUs, reflecting a baseline or affectively flat facial configuration. In this study, the Py-Feat software was used to automatically code AUs from video frames (27). Smiling was quantified by the presence of AU12, and social smiling was operationalized as the simultaneous occurrence of AU12 activation and center gaze. This operationalization of social gaze was adapted from prior POV-based studies in children with autism (28).

Finally, speech features were extracted using Whisper, an open-source deep learning model for automatic speech recognition trained on a large-scale multilingual and multitask dataset. Whisper has demonstrated high performance in Turkish automatic speech recognition tasks, making it a suitable tool for analyzing speech features in this study (29). Transcripts were generated with word-level timestamps and aligned to the interview structure. Question–answer boundaries were defined using the *end of the interviewer’s question* (question-end time) as the reference point. Response latency was computed as the time (ms) from this question-end to the onset of the participant’s first frame in the subsequent answer segment. Speech/silence segmentation relied on voice-activity detection (VAD) with 30-ms frames and 10-ms hops; non-speech frames were identified by the VAD and very short gaps (<150 ms) were merged to avoid spurious pauses. The *silence ratio* was defined as the proportion of non-speech frames within each answer segment (leading/trailing silences outside the answer boundaries were excluded). Word count was computed on participant speech only. These features (response latency, silence ratio, word count) were then used in subsequent statistical and machine-learning analyses. All features were derived automatically. Manual spot-checks on a random subset confirmed alignment between visual overlays and extracted values across all domains.

### Statistical analysis

All statistical analyses were conducted using IBM SPSS Statistics (Version 27, IBM Corp, Armonk, NY) and Python (Version 3.9) with the SciPy library. The normality of continuous variables was assessed using the Shapiro–Wilk test. For normally distributed data, independent-samples *t* tests were used to compare group means between the MDD and control groups. For non-normally distributed data, Mann–Whitney *U* tests were applied. Categorical variables were compared using chi-square tests.

Because multiple, conceptually related outcomes were tested, we controlled the family-wise error rate within behavioral domains rather than across all individual variables. Specifically, the 15 features were organized *a priori* into five domains based on theoretical and measurement considerations: (i) gaze-related measures (center/”eye contact”, right, left; *m*(gaze)=3), (ii) facial affect measures (smiling duration [AU12], happy faces, neutral faces, social smiling [AU12 with center gaze]; *m*(affect)=4), (iii) ocular physiology (blink count, blink duration, eye openness; *m*(ocular)=3), (iv) head movement (total head movements, rapid head movements; *m*(head)=2), and (v) speech-derived features (response latency, silence ratio, word count; *m*(speech)=3). Within each domain, a Bonferroni correction was applied as α_domain = .05/*m*_domain, yielding the following adjusted thresholds used for primary inference:

➢ Gaze: α = .05/3 ≈.0167

➢ Facial affect: α = .05/4 = .0125

➢ Ocular physiology: α = .05/3 ≈.0167

➢ Head movement: α = .05/2 = .0250

➢ Speech: α = .05/3 ≈.0167

This domain-wise control limits Type I error where outcomes are correlated within the same construct, while avoiding the over-conservatism of correcting across all 15 endpoints simultaneously. In the Results, we report exact *P* values; findings surpassing the domain-specific α thresholds are denoted as significant after correction, and findings with *P* <.05 but ≥ α_domain are described as nominal (uncorrected) and interpreted cautiously.

With a total sample of n=85 (MDD = 44, HC = 41), two-sided tests at α=.05 provide 80% power to detect a standardized mean difference of approximately Cohen’s d ≈ 0.61 (≈ r ≈ 0.29, medium). Under the domain-wise thresholds used in this study, the 80% power minimum detectable effects are d ≈ 0.67 for α=.025 (head movement), d ≈ 0.70 for α=.0167 (gaze/ocular/speech), and d ≈ 0.72 for α=.0125 (facial affect) (corresponding r ≈ 0.32–0.34). Thus, the study is well-powered to detect medium-to-large effects, while smaller effects (d < ~0.6) may be underpowered.

### Machine learning analysis

In addition to group-level comparisons, machine learning methods were employed to evaluate the classification performance of behavioral features in distinguishing between patients with MDD and healthy controls. We implemented a supervised learning pipeline and evaluated several algorithms (e.g., ExtraTrees, Random Forests, Gradient Boosting, k-nearest neighbors, and support vector machines

To enhance generalizability while preventing information leakage, recursive feature elimination (RFE) and all preprocessing steps were performed within each training fold of the cross-validation procedure. RFE systematically removed features with the lowest importance at each iteration until an optimal subset of predictors was identified.

To ensure robust evaluation with limited data, model performance was assessed using nested leave-one-out cross-validation (LOOCV). In the inner loop, RFE and hyperparameter tuning were conducted using only the training data, while in the outer loop the held-out participant served exclusively for testing. To improve calibration and interpretability, probabilistic outputs were adjusted in a fold-specific manner using either Platt scaling or isotonic regression on the training fold only. Performance metrics included accuracy, precision, recall (sensitivity), specificity, F1-score, and the area under the receiver operating characteristic curve (ROC-AUC). Feature importance scores were also examined to identify the most discriminative behavioral markers.

### Ethical considerations

The study protocol was reviewed and approved by the Non-Interventional Clinical Research Ethics Committee of Zonguldak Bülent Ecevit University (approval number: 2024/21). Written informed consent was obtained from all participants prior to enrollment. All data were anonymized, and confidentiality was strictly maintained throughout the study.

## Results

### Statistical analysis

The demographic characteristics of the groups were comparable. The MDD group consisted of 24 women and 20 men, while the control group included 21 women and 20 men, with no significant difference in sex distribution (*χ²*(1)=0.05, *P* = .829). The mean age of patients with MDD was 37.81 years, compared with 37.17 years in the control group, again showing no significant difference (*t*(83)=0.238, *P* = .813).

Prior to group comparisons, the distribution of variables was examined using both Kolmogorov–Smirnov and Shapiro–Wilk tests. Only center gaze and word count met the criteria for normal distribution across both groups, and these were analyzed using independent-samples *t*-tests. All other behavioral features deviated significantly from normality and were therefore examined using Mann–Whitney U tests. This analytical approach ensured that the most appropriate statistical methods were applied based on the distributional characteristics of each variable.

Happy facial expressions were markedly reduced in patients with MDD (mean rank=34.53) relative to controls (mean rank=52.09), Mann–Whitney U = 529.5, Z=–3.29, *P* = .001. This difference survived Bonferroni correction (α=.0125) and thus represents a robust group-level effect.

Clear between-group differences were found in multiple facial affect parameters, with some effects surviving Bonferroni correction and others reaching only nominal significance. Smiling duration was significantly shorter in the MDD group (mean rank=35.75) compared with controls (mean rank=50.78), Mann–Whitney U = 583.0, Z=–2.82, *P* = .005. This effect reached significance at the corrected threshold (corrected α=.0125).

Social smiling, defined as the simultaneous occurrence of smiling and center gaze, was also lower in the MDD group (mean rank=37.09) compared with controls (mean rank=49.34), Mann–Whitney U = 642.0, Z=–2.33, *P* = .020. This result achieved nominal significance but did not survive the corrected threshold.

Clear group differences emerged in gaze directed toward the interviewer, whereas lateral gaze parameters showed weaker or nonsignificant effects. Patients with MDD spent significantly less time fixating on the interviewer’s face compared with healthy controls (M = 13.51, SD = 5.75 vs M = 19.22, SD = 3.78). This difference was highly robust, Welch’s *t*(74.87) = –5.44, *P* <.001, 95% CI [–7.80, –3.62], Cohen’s *d* ≈ –1.16, and survived Bonferroni correction for the gaze domain (corrected α = .0167).

For rightward gaze, patients with MDD displayed longer gaze durations (mean rank=48.89) relative to controls (mean rank=36.68), Mann–Whitney U = 643.0, Z=–2.28, *P* = .023. Although this difference reached nominal significance at the uncorrected threshold (*P* <.05), it did not survive the domain-specific Bonferroni adjustment. Leftward gaze duration did not differ between groups (mean rank=46.83 vs 38.89; U = 733.5, *P* = .138).

Finally, neutral facial expressions were more prevalent in the MDD group (mean rank=49.27) than in controls (mean rank=37.09), Mann–Whitney U = 626.0, Z=–2.42, *P* = .015. This effect was nominally significant but did not remain after Bonferroni correction.

Group comparisons of ocular features revealed differences in blink-related parameters, whereas eye openness did not vary between groups. For blink count, patients with MDD (mean rank=46.42) did not differ significantly from controls (mean rank=39.33), Mann–Whitney U = 751.5, Z=–1.32, *P* = .185. Blink duration was longer in the MDD group (mean rank=48.73) compared with controls (mean rank=36.85), Mann–Whitney U = 650.0, Z=–2.22, *P* = .026. This effect reached nominal significance (*P* <.05) but did not survive Bonferroni correction for the ocular domain (corrected α=.0167). Eye openness did not differ significantly between groups (mean rank=44.57 vs 41.32), Mann–Whitney U = 833.0, Z=–0.61, *P* = .543.

No significant between-group differences were found for head movement parameters. For total head movements, the MDD group (mean rank=45.09) and controls (mean rank=40.76) did not differ significantly, Mann–Whitney U = 810.0, Z=–0.81, *P* = .418. Similarly, rapid head movements were comparable across groups (mean rank=42.49 vs 43.55), Mann–Whitney U = 879.5, Z=–0.20, *P* = .843. Both effects were far from significance and did not approach the corrected α threshold for the head movement domain (α=.025).

No significant group differences were observed for speech-derived features. For word count, patients with MDD (M = 43.02, SD = 10.40) and controls (M = 45.89, SD = 9.29) did not differ significantly, *t*(83)=–1.34, *P* = .183, 95% CI [–7.13, 1.38]. Response latency (time to first word) was comparable between groups (mean rank=44.50 vs 41.39), Mann–Whitney U = 836.0, Z=–0.58, *P* = .561. Similarly, silence ratio did not differ significantly between patients (mean rank=43.25) and controls (mean rank=42.73), Mann–Whitney U = 891.0, Z=–0.10, *P* = .923. None of these measures approached significance under the uncorrected threshold, and therefore no effects survived Bonferroni adjustment for the speech domain (α=.0167). Statistically significant and nominally significant features are provided in **Table 1**. The comprehensive version of this table (which includes non-significant features) can be found in **Table 2**.

**Table 1:** Summary of statistically significant and nominally significant features across domains.

Measure	Test	Statistic	P-value	Bonferroni (domain)	Group difference (MDD vs. HC)
Center gaze	Welch’s t-test	t(74.87) = –5.44	<.001	Significant after correction	MDD < HC
Right gaze	Mann–Whitney U	U = 643.0, Z = –2.28	.023	Nominally significant only	MDD > HC
Smiling duration	Mann–Whitney U	U = 583.0, Z = –2.82	.005	Significant after correction	MDD < HC
Happy face	Mann–Whitney U	U = 529.5 Z = –3.29	.001	Significant after correction	MDD < HC
Social smiling	Mann–Whitney U	U = 642.0, Z = –2.33	.020	Nominally significant only	MDD < HC
Neutral face	Mann–Whitney U	U = 626.0, Z = –2.42	.015	Nominally significant only	MDD > HC
Blink duration	Mann–Whitney U	U = 650.0 Z = –2.22	.026	Nominally significant only	MDD > HC

**Table 2:** Group comparisons of behavioral features between MDD and control groups.

Domain	Measure	Test	Statistic	P-value	Bonferroni (domain)	Group difference (MDD vs. HC)
Gaze	Center gaze	Welch’s t-test	t(74.87) = –5.44	<.001	Significant after correction	MDD < HC
	Right gaze	Mann–Whitney U	U = 643.0, Z = –2.28	.023	Nominally significant only	MDD > HC
	Left gaze	Mann–Whitney U	U = 733.5, Z = –1.48	.138	Not significant	No difference
Facial affect	Smiling duration	Mann–Whitney U	U = 583.0, Z = –2.82	.005	Significant after correction	MDD < HC
	Happy face	Mann–Whitney U	U = 529.5 Z = –3.29	.001	Significant after correction	MDD < HC
	Social smiling	Mann–Whitney U	U = 642.0, Z = –2.33	.020	Nominally significant only	MDD < HC
	Neutral face	Mann–Whitney U	U = 626.0, Z = –2.42	.015	Nominally significant only	MDD > HC
Ocular physiology	Blink count	Mann–Whitney U	U = 751.5, Z = –1.32	.185	Not significant	No difference
	Blink duration	Mann–Whitney U	U = 650.0 Z = –2.22	.026	Nominally significant only	MDD > HC
	Eye openness	Mann–Whitney U	U = 833.0, Z = –0.61	.543	Not significant	No difference
Head movement	Total head movement	Mann–Whitney U	U = 810.0, Z = –0.81	.418	Not significant	No difference
	Rapid head movement	Mann–Whitney U	U = 879.5, Z = –0.20	.843	Not significant	No difference
Speech	Word count	Independent t-test	t(83) = –1.34	.183	Not significant	No difference
	Time to first word	Mann–Whitney U	U = 836.0, Z = –0.58	.561	Not significant	No difference
	Silence ratio	Mann–Whitney U	U = 891.0, Z = –0.10	.923	Not significant	No difference

### Machine learning classification results

To complement the group-level comparisons, machine learning analyses were conducted to evaluate the predictive value of multimodal behavioral features for distinguishing individuals with MDD from healthy controls. Several supervised learning methods were initially tested, including tree-based algorithms (random forests, gradient boosting), nearest neighbor classifiers, and support vector machines. Among these, the ExtraTrees algorithm demonstrated superior and more stable performance across evaluation metrics and was therefore selected as the primary model (30).

**Figure 5:**
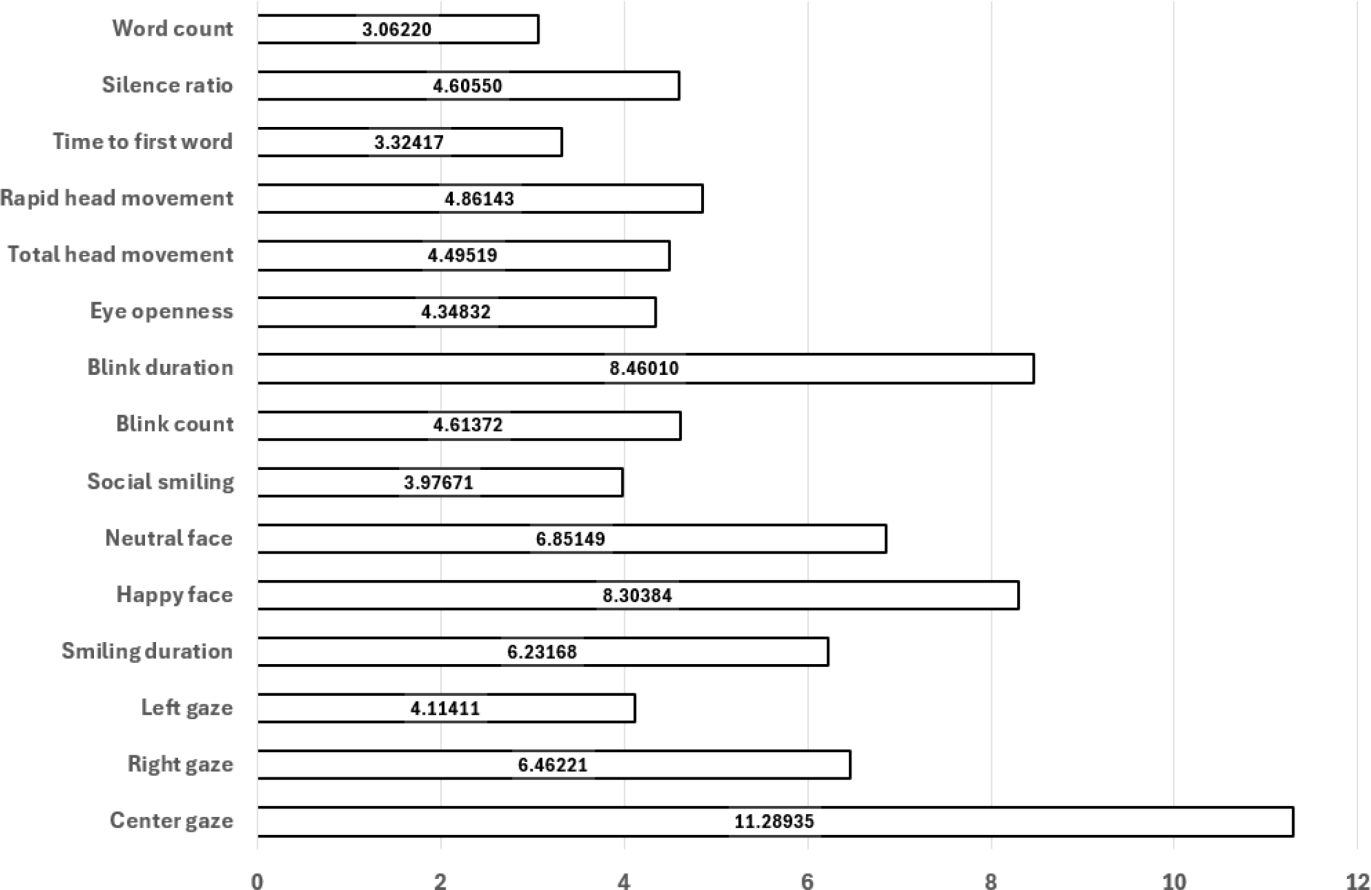
Model-averaged feature importances; higher values indicate greater contribution to classification.

Prior to model training, the feature importance values were calculated. This was achieved through obtaining importance values for each supervised learning method described above. The Gini impurity-based feature importances are automatically calculated through each fitting procedure, as part of most supervised learning models’ supported programming libraries. Once feature importances were calculated, they were averaged across each supervised learning model, yielding a robust perspective of importance for each feature. Importance results are provided in the figure below. (**Figure 5**) Recursive feature elimination (RFE) was applied to iteratively remove features with the lowest importance scores, yielding a reduced subset of predictors with higher generalizability. This procedure aimed to minimize overfitting, particularly given the relatively modest sample size.

Model validation was performed using the leave-one-out cross-validation (LOOCV) strategy, which maximizes data utilization by iteratively training on all participants except one and testing on the held-out individual. This approach provides a robust estimate of model performance in small-sample studies.

The ExtraTrees classifier demonstrated strong performance, achieving an AUC of 0.89, accuracy of 84.7%, precision of 81.6%, sensitivity (recall) of 90.9%, specificity of 78.0%, and an F1 score of 86%. The model also achieved an AUC score of 0.89, indicating a high overall ability to distinguish between the two classes.

Performance with confidence intervals. Using pooled outer-fold test predictions from LOOCV, proportion metrics were reported with Wilson 95% CIs: accuracy 84.7% (75.5–90.8), precision 81.6% (72.1–88.4), sensitivity 90.9% (82.9–95.4), and specificity 78.0% (68.1–85.5) (n=85). The F1-score was 86.0% (77.1–91.8), estimated via stratified bootstrap. The ROC–AUC was 0.89 (95% CI 0.82–0.96), with the interval computed using the Hanley & McNeil (1982) normal approximation based on the pooled outer-fold predictions (positives N_1_=44 MDD, negatives N_2_=41 HC) (31). These intervals quantify uncertainty using held-out (outer-fold) test outputs only.

**Figure 6:**
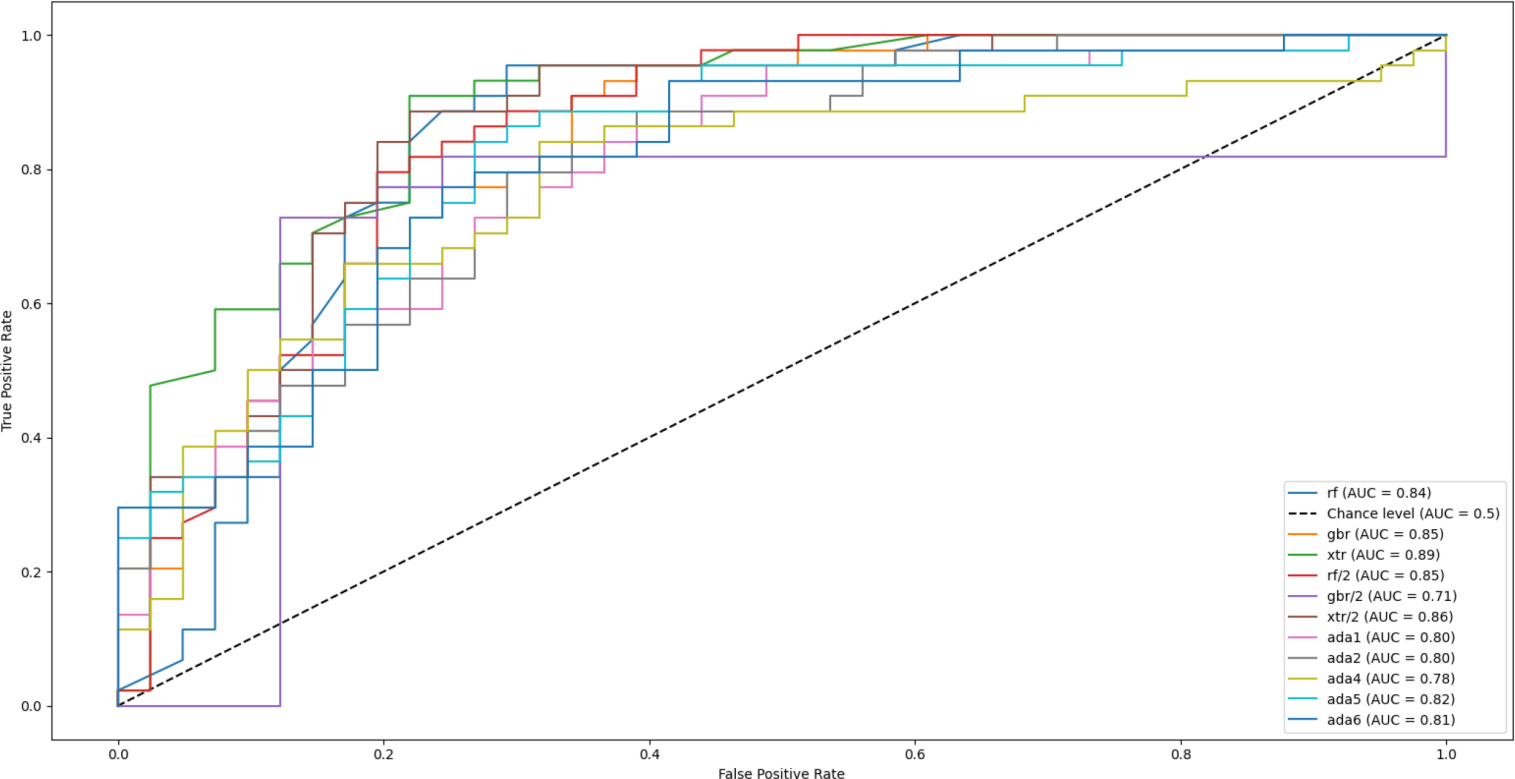
Receiver operating characteristic (ROC) curves for multiple supervised machine learning models used to classify major depressive disorder (MDD) and healthy controls. The *ExtraTrees* classifier achieved the highest performance (AUC = 0.89), followed by AdaBoost (AUC = 0.83) and Random Forest (AUC = 0.81). The diagonal dashed line represents chance-level performance.

**Figure 6** presents the Receiver Operating Characteristic (ROC) curve for the classification task addressed in this study. The curve illustrates the relationship between the true positive rate and the false positive rate across varying classification thresholds, thereby providing an overall view of the model’s discriminative performance. The area under the curve (AUC) is used as a quantitative indicator of performance, where higher values denote stronger separation between the positive and negative classes. As shown, multiple ROC curves have been obtained, each by a different supervised learning model. The Extra Trees algorithm (curves labeled ‘xtr’ and ‘xtr/2’, green and brown, respectively) can be observed to be outperforming the remaining models in effectively distinguishing between the two classes, confirming its reliability in the context of the problem. The support vector machines and nearest neighbor methods were excluded from this specific analysis as they were already eliminated from the pool of potential models. Extra Trees introduces more randomization by selecting split thresholds at random, which helped reduce overfitting and improved generalization, especially on noisy data. Additionally, the increased randomness led to greater model diversity, resulting in slightly better predictive performance compared to methods like Random Forests and Gradient Boosting.

## Discussion

### Principal findings

This study demonstrated that behavioral features extracted from point-of-view (POV) video recordings during clinical interviews can reliably distinguish patients with major depressive disorder (MDD) from healthy controls. To our knowledge, this is the first study to use clinician-worn point-of-view (POV) video to capture multimodal behavioral signals during real clinical interviews with patients diagnosed with MDD. In our results, although the two groups were comparable in age and sex, significant differences emerged in gaze and affective expression. Specifically, patients with MDD exhibited shorter durations of smiling duration, center gaze and happy facial expressions compared to controls. These three parameters remained significant even after Bonferroni correction for multiple comparisons, highlighting their robustness as potential behavioral markers of depression.

Additional group-level differences were observed at nominal significance levels, including reduced social smiling (simultaneous smiling and center gaze), and prolonged neutral facial expressions among patients with MDD. Right gaze and blink duration were also higher in the MDD group, though these findings did not survive correction for multiple testing. Together, these results suggest that both gaze-related and affective cues may reflect disruptions in social engagement and emotional expressivity characteristic of depression.

In contrast, head movement parameters (total and rapid head movements) and speech-derived features (response latency, silence ratio, and word count) did not differ significantly between groups.

Beyond group-level comparisons, the application of machine learning further supported the diagnostic value of these multimodal features. The ExtraTrees classifier—optimized using recursive feature elimination and validated through leave-one-out cross-validation—demonstrated strong overall performance (accuracy = 84.7%, AUC = 0.89, F1-score = 86%). These results suggest that multimodal behavioral data, collected through a low-burden point-of-view (POV) methodology, provide a meaningful contribution to the objective and scalable assessment of depression.

### Comparison with prior work

Our findings align with prior research indicating that patients with MDD exhibit reduced gaze toward social partners and diminished positive affective expressions. Previous eye-tracking studies in laboratory settings have consistently reported shorter fixation durations and reduced eye contact in individuals with depression, which have been interpreted as behavioral markers of social withdrawal and negative self-referential bias (32–34). Eye contact, broadly defined as the mutual exchange of gaze between two individuals, is a cornerstone of social communication. It facilitates conversation initiation, turn-taking, and the regulation of arousal during interpersonal interactions, while also allowing individuals to track the attentional and emotional states of their partners (35–38). Developmental research further underscores its importance, showing that infants preferentially orient to direct gaze and that mutual gaze plays a key role in attachment and bonding (39, 40).

Against this background, the reduced center gaze duration observed in our study supports the notion that depression disrupts core mechanisms of social engagement. Interestingly, patients with MDD in our sample also showed a tendency toward prolonged rightward gaze. Alghowinem et al. similarly reported that depressed individuals spent more time directing their head and gaze to the right side during stationary camera recordings, which was interpreted as a behavioral manifestation of gaze aversion and reduced willingness to engage in social interaction (41). Although this lateralization effect did not remain significant after correction in our data, the pattern may nonetheless reflect a subtle form of social disengagement or avoidance of direct interpersonal contact.

Eye blinking is not merely a reflexive behavior but is also regulated by the central nervous system and influenced by neurotransmitters such as dopamine (42). Blink rate has been proposed as an indirect marker of dopaminergic activity and is known to vary with attentional demands, emotional state, and mental tension (43). However, findings regarding the association between blink rate and depression remain inconsistent across studies. Mackintosh et al. reported that patients with depression exhibited higher blink rates in video-based assessments, which subsequently decreased following treatment (44). Similarly, Lee et al. observed increased blink frequency particularly in late-life depression (45). Another study employing OpenCV and convolutional neural networks (CNNs) to detect blinking found a weak but positive correlation between blink rate and depression severity (R²≈0.035) (46). In our study, patients with MDD also showed numerically higher blink counts compared with controls, although this difference did not reach statistical significance.

Beyond blink frequency, additional ocular parameters such as blink duration and eye openness provide complementary information about oculomotor control and affective state. Prior studies have used webcam recordings during the presentation of emotionally valenced stimuli (e.g., happy or sad videos) and applied the Eye Aspect Ratio (EAR) to categorize eye states as open, partially closed, or closed (47). These works suggested that reduced eye openness and a “partially closed eye” appearance may be more frequently observed in individuals with depression, potentially serving as behavioral markers of affective blunting. Furthermore, it has been proposed that combining blink dynamics with other ocular signals, such as gaze direction or pupil diameter, could improve the reliability of depression-related biomarkers. Ramalho et al. reported that patients with depression showed decreased eye openness and prolonged blink duration when measured via EAR-based analysis from webcam recordings (47).

In line with these findings, our study revealed that eye openness did not differ between groups, but blink duration was significantly prolonged in the MDD group. One possible explanation for this pattern is that extended eyelid closure may reflect psychomotor retardation, a common clinical feature of depression, manifesting as slowed or prolonged motor acts. Alternatively, longer blinks could represent subtle disengagement from social interaction, consistent with broader withdrawal tendencies observed in depression. However, given the mixed results in the literature, further studies incorporating multimodal ocular measures will be necessary to clarify the mechanisms underlying these differences.

Reduced smiling and diminished happy facial expressions are well-documented features of depression, repeatedly confirmed across behavioral and observational studies (48, 49). In depressed individuals, the frequency, intensity, and duration of smiling and happy facial expressions are consistently lower compared with healthy controls (50, 51), with spontaneous (nonvolitional) smiles being particularly reduced (52, 53). By contrast, research on neutral facial expressions in depression remains relatively scarce and lacks a clear consensus. For instance, Lee et al. reported reduced neutral expressions in a simulation paradigm; however, participants in that study deliberately exaggerated sadness-related expressions, which may confound interpretation (54). Similarly, Wang et al. using stationary cameras and instructed emotion tasks, found a general reduction in facial variability among depressed individuals, suggesting less flexibility in affective expression (55).

In our study, both smiling and happy expressions were attenuated in the MDD group, while neutral expressions were increased. These findings are consistent with previous evidence identifying anhedonia and affective blunting as core behavioral manifestations of depression. These results suggest that reduced positive affect—captured through multimodal visual markers such as smiling and gaze engagement—may reflect diminished reward sensitivity and social motivation. In parallel, the clinical management of treatment-resistant depression (TRD) remains an active area of debate and development (56), and recent studies report pharmacological avenues targeting anhedonia and affective functioning (57) as well as related cross-diagnostic evidence (58). These trends underscore that, beyond treating TRD, objective markers are needed to identify, monitor, and compare treatment response across settings.

Our POV-based markers quantify state-level expressions of positive affect and social engagement that dovetail with trait-level affective temperaments described in contemporary spectrum models. Temperament research indicates that these genetically influenced dispositions underlie mood, substance-use, and risk-taking disorders; shape clinical presentation, course, and treatment response (especially in bipolar disorder and MDD); and relate to insight and role functioning across phases (59, 60). Within this framework, our results align with temperamental liabilities linked to anhedonia/affective blunting (e.g., depressive/cyclothymic poles), while also suggesting that hyperthymia—often discussed as protective—may correspond to greater smiling and sustained gaze engagement. Taken together, integrating objective, POV-derived behavioral biomarkers with pharmacological evidence and temperament assessments strengthens the translational potential of digital phenotyping.

The operationalization of “social smiling” as the temporal overlap of forward gaze and smiling provides an ecologically meaningful index of socially contingent affective responses. While this construct has been primarily studied in POV-based research on children with autism (28), it has not, to our knowledge, been systematically investigated in adult psychiatric populations, highlighting the novelty of the present study. In our sample, the duration of social smiling was numerically reduced in patients with MDD and reached nominal significance at the uncorrected level. However, this effect did not survive Bonferroni correction, indicating that the finding should be interpreted with caution. Nevertheless, the trend toward diminished social smiling is consistent with the broader literature on depression, which emphasizes reductions in both eye contact and positive affective displays.

Prior research has consistently associated depression with reduced or slowed head movement dynamics. Alghowinem et al. examined 30 patients with severe depression and 30 healthy controls using stationary camera recordings, extracting head pose changes at 10-frame intervals. Their results indicated markedly slower head movements, characterized by lower pitch and yaw velocities, and fewer directional changes overall, reflecting generalized motor slowing (41). Similarly, Kacem et al. employed the ZFace toolkit to identify 49 facial landmarks and reported decreased head motion amplitude in participants with more severe depressive symptoms (61).

In contrast to these findings, our study did not observe significant group differences in total or rapid head movements. Several methodological factors may account for this discrepancy. First, our sample comprised individuals meeting diagnostic criteria for MDD but not necessarily exhibiting severe symptomatology, and depression severity was not modeled continuously. Second, unlike prior studies that relied on stationary laboratory cameras, our recordings were collected from naturalistic clinical interviews using a POV setup worn by the interviewer. This configuration captures ecologically valid interpersonal exchanges but may introduce variability related to interactive dynamics. For instance, participants in the control group tended to maintain a more sustained center gaze toward the interviewer, potentially reducing the need for larger head movements. Conversely, subtle nods or posture adjustments during conversation might have been more frequent but less pronounced in depressed participants, making quantitative differences harder to detect.

Taken together, these findings suggest that head movement patterns may be sensitive to both methodological context and depression severity. Future research integrating continuous severity measures and multimodal motion tracking could help clarify whether diminished head movement represents a stable biomarker or a context-dependent behavioral correlate of depression.

Alterations in speech patterns have long been recognized as characteristic features of depressive states. Prior studies have demonstrated that individuals with depression tend to speak more slowly, with lower vocal intensity, reduced prosodic variation, and longer pauses compared with healthy controls. For example, Cummins et al. reported that pause duration was significantly prolonged among English-speaking participants with depression, although this effect was not consistently replicated across other languages, suggesting potential linguistic or cultural modulation of speech-related markers (62). Similarly, Yamamoto et al. analyzed unstructured interview recordings and found that patients with depression exhibited slower speech rate, longer response latencies, and extended pauses relative to nondepressed participants (63).

In our study, although the MDD group produced fewer words on average than healthy controls, this difference did not reach statistical significance. This finding may indicate that while verbal productivity shows a downward trend in depression, the magnitude of the effect can vary depending on contextual factors such as task structure, language, or interview dynamics. Given that our data were collected during naturalistic clinical interviews rather than constrained reading or speech tasks, subtle alterations in temporal features of speech may have been less pronounced.

With the rapid advancement of artificial intelligence, machine learning (ML) has become an increasingly valuable tool for identifying complex behavioral and biological markers of psychiatric disorders. Traditional diagnostic methods in psychiatry rely heavily on clinician-administered interviews and self-reported questionnaires, which can be subjective and prone to bias. In contrast, ML-based approaches enable the extraction of objective, high-dimensional features from multimodal data such as facial expressions, speech, physiological signals, and self-reports, offering new pathways for early detection and personalized treatment of depression. Recent work introduced Clinical 15, a machine learning model trained on multimodal data from primary care settings, which achieved a balanced accuracy of 88.2% and demonstrated the feasibility of integrating biological, physiological, and self-reported information for differential diagnosis (64). Similarly, a deep learning framework was proposed that combines facial video and audio modalities using spatiotemporal attention and graph convolutional networks to enhance multimodal feature fusion, achieving robust performance in automatic depression detection (65). Complementing these findings, it has been emphasized that incorporating behavioral and physiological signals into ML-based frameworks can outperform traditional rating scales in predicting depressive states, underscoring the importance of multimodal integration for improving clinical validity (66). In this context, our study adds novel evidence by demonstrating that a wearable, first-person (POV) system can achieve a high classification accuracy (84.7%) and AUC of 0.89, comparable to or exceeding performance metrics reported in prior multimodal ML studies. Importantly, by using a low-burden and ecologically valid setup, our approach bridges the gap between controlled laboratory research and real-world clinical application, underscoring the potential of wearable ML-assisted systems for scalable, objective assessment of depression in everyday contexts. Beyond its technical contribution, this study also introduces a novel methodological framework by integrating multiple behavioral domains—facial affect, gaze behavior, head movement, and speech—captured during authentic clinician–patient interactions. Unlike webcam-based or stationary laboratory recordings, our approach collects data from real clinical interviews without disrupting their natural flow, providing a more genuine representation of social and affective behavior in depression. This integration of ecological validity with multimodal computational analysis represents an important step toward objective, clinically applicable assessment methods for depressive disorders.

Finally, responsible deployment of POV-based capture in clinical settings requires explicit attention to ethics and data protection. Because POV capture can incidentally record bystanders and sensitive contexts, its use should follow privacy-by-design: clear consent and user control; purpose-limited collection; data minimization with on-device or ephemeral processing and retention of derived features rather than raw video when feasible; de-identification/pseudonymization, encrypted storage and role-based access; time-bound retention and deletion; and site-level governance and transparency. These safeguards, aligned with contemporary frameworks are essential for any clinical deployment. If these privacy conditions can be met in practice, POV capture may be a feasible option for clinical research and—subject to context, clear opt-out pathways, and human oversight—could potentially be considered for clinician use at the point of care. These practices align with recent calls in the affective computing literature to ground deployments in privacy-by-design and societal value (67).

### Limitations

Several limitations should be acknowledged. First, although the sample encompassed participants across a spectrum of depressive severity—including some severe cases—the recruitment was not restricted solely to severe MDD. Diagnostic classification was based on SCID-5 interviews combined with Beck Depression Inventory (BDI) scores above 20, leading to a binary grouping (MDD vs. control). Consequently, we did not model depression severity as a continuous variable or examine stratified performance across severity levels. Future studies should incorporate continuous BDI scores as predictive features to explore whether accounting for symptom intensity enhances model discrimination. While restricting the sample to more severe cases may yield higher classification accuracy, prior work indicates that machine learning models often exhibit lower accuracy when trained on heterogeneous, real-world clinical populations, highlighting the trade-off between performance and ecological generalizability.

Second, this single-site study enrolled 85 participants—a sample size comparable to prior depression-recognition work yet still modest for high-dimensional multimodal analyses. To establish robustness and transportability, external validation in independent, demographically diverse cohorts is essential; multi-center external validation studies are planned.

Third, our speech features were restricted to basic linguistic and timing measures (e.g., word count, response latency, silence ratio), which may overlook relevant acoustic–prosodic cues. Future studies could incorporate F0/pitch, intensity, jitter–shimmer, MFCCs, spectral/temporal prosody, and turn-taking dynamics to potentially enhance sensitivity and interpretability.

In addition, the wearable POV setup—central to the study’s ecological validity—introduces variability related to participant motion, lighting conditions, and camera alignment, which may have introduced minor measurement noise despite averaging across four 30-second conversational segments. Finally, some automatically extracted features (e.g., gaze and facial action units) depend on computer vision pipelines that are inherently susceptible to tracking errors. Implementing confidence-weighted smoothing, temporal filtering, or hybrid sensing methods in future studies could improve reliability.

For real-world scalability, future work could (a) pilot a mobile-health workflow that ships a lightweight POV device to participants (with consented upload via a secure app) to replicate our features in natural home settings, and (b) develop a telepsychiatry-compatible variant using a brief left–center–right gaze and affect calibration plus domain adaptation to handle off-axis views and lighting. Such studies would help ensure generalizability and calibration across settings, while accelerating privacy-preserving, real-world deployment.

### Conclusions

This study demonstrates that multimodal behavioral features—captured through wearable point-of-view (POV) glasses during clinical interviews—can reliably differentiate individuals with major depressive disorder (MDD) from healthy controls. Visual markers, particularly reduced center gaze and diminished positive affect, emerged as robust indicators of depression, aligning with established theories of social withdrawal and affective blunting. Importantly, a machine learning classifier trained on features derived from naturalistic audiovisual recordings achieved high diagnostic accuracy (84.7%) and strong discriminative performance (AUC = 0.89).

These findings underscore the potential of wearable, low-burden technologies to facilitate objective, scalable, and ecologically valid assessments of depression in real-world clinical settings. By capturing authentic clinician–patient interactions, the proposed framework advances the integration of behavioral signal processing and machine learning in psychiatric evaluation.

## Data Availability

The raw data supporting the conclusions of this article will be made available by the authors, without undue reservation.
